# Determinants and spatial patterns of adult overweight and hypertension in a high HIV prevalence rural South African population

**DOI:** 10.1016/j.healthplace.2012.09.001

**Published:** 2012-11

**Authors:** Jiachen Zhou, Mark N. Lurie, Till Bärnighausen, Stephen T. McGarvey, Marie-Louise Newell, Frank Tanser

**Affiliations:** a Department of Epidemiology, Public Health Program, Brown University, Providence, RI, USA; b Africa Centre for Health and Population Studies, University of KwaZulu-Natal, P.O. Box 198, Mtubatuba 3935, South Africa; c Department of Global Health and Population, Harvard School of Public Health, Boston, MA, USA; d Centre for Paediatric Epidemiology and Biostatistics, University College London Institute of Child Health, UK

**Keywords:** Overweight, Hypertension, Disease clustering, Geographical information systems

## Abstract

We conducted a large population-based survey among adults measuring weight, height, and blood pressure nested within an HIV survey in rural KwaZulu-Natal, South Africa, to identify and characterize clusters of overweight and hypertension in a typical rural African population and to explore whether geographic clusters can be accounted for by established individual-level risk factors. 58.4% of the participants were overweight and 22.6% were hypertensive. One cluster of high prevalence of overweight (RR=1.50, *p*<0.001) was identified using Kulldorff spatial scan statistic as the most likely cluster, whereas a low-risk cluster was identified in the nearby high-density settlement area (RR=0.62, *p*<0.05). No geographic clusters of hypertension were identified. After controlling for age, sex, educational attainment, household wealth, marital status, place of residence, and HIV status, no spatial clustering of overweight remained. The results provided clear evidence for the localized clustering of overweight. Identification of clustering of chronic disease could provide additional insights into the prevention and control for the rural South African population.

## Highlights

► Determinants and spatial patterns of chronic diseases in rural South Africa. ► Localized clustering of overweight identified, but no clustering for hypertension. ► Additional insights into chronic disease control for rural South Africa were provided.

## 1 Introduction

Once considered a health burden confined to high income countries, the prevalence of overweight and obesity is now increasing in low- and middle-income countries (WHO, 2000). In South Africa, overweight and obesity are prevalent among almost all population and age groups, especially in the rural areas (Bourne et al., 2002; Mayosi et al., 2009; Mollentze et al., 1995; Vorster, 2002). Already, non-communicable diseases related to overweight and obesity, such as heart disease, diabetes, and stroke, together constitute the second most common cause of death among adult South Africans (Bradshaw et al., 2003). The prevalence of obesity (body mass index (BMI)≥30) in adult men and adult women (over the age of 15 years), respectively, is 9% and 27%, according to the South African Demographic and Health Survey conducted in 2003 (Department of Health, 2007).

Most studies and interventions of obesity have focused on determinants at the individual level (Black and Macinko, 2008; Suastika, 2006). These approaches targeting individuals may not be sufficient to prevent or curb the increasing trends of obesity (Yancey et al., 2004). Lately, some researchers, mostly from developed countries, have started studying the wider neighborhood-level characteristics of body weight, hoping to discover new approaches to better understand and curb the continuing obesity epidemic (Black and Macinko, 2008; Yancey et al., 2004). Studies from developed countries have suggested that low community-level socioeconomic resources are related to high prevalence of obesity (Black and Macinko, 2008). However, whether findings from studies conducted in highly industrialized regions can be applied to low/middle income countries remains uncertain. For example, although the availability of green space had been linked to decreased adult BMI, a recent study conducted in Cairo, Egypt, did not find the same significant association (Mowafi et al., 2012). Hypertension has also been found to be associated with some modifiable socioeconomic determinants, such as education and occupation (Grotto et al., 2008), although it is less studied at the population level.

In the South African context, increases in total fat and animal protein intake of affluent black South Africans were associated with increases in BMI and in total serum cholesterol (Vorster, 2002). Ambulation was found to reduce the risk of obesity, while access to a motor vehicle was associated with increased adiposity levels among rural black South African women (Cook et al., 2008). Besides, hypertension has been reported to be an important health challenge for the mining industry in South Africa (Hessel, 1985; Maepe and Outhoff, 2012).

To our knowledge, no studies have documented community-level socioeconomic determinants of obesity or hypertension in a rural South African community. To inform the design of disease prevention and intervention programs, it is helpful to understand factors that could contribute to geographic variations in their respective prevalence. Geographic analysis has been used to identify and characterize clustering of non-communicable diseases, including overweight and obesity, mostly in developed countries (Edwards and Clarke, 2009; Ford et al., 2005; Pouliou and Elliott, 2009). In South Africa, geographic analysis has been used to detect and analyze the patterns of HIV/AIDS cases (Tanser et al., 2009), tuberculosis cases (Beyers et al., 1996; van Rie et al., 1999), and the distribution of healthcare points and the effect of community-based tuberculosis treatment on increased access to nearest treatment supervision point (Tanser and Wilkinson, 1999; Wilkinson and Tanser, 1999).

In this study, we use population-based data to identify and characterize spatial clusters of overweight (BMI≥25) and hypertension in a typical rural South African population with high HIV prevalence, and to explore whether these clusters, of high or low prevalence, could be accounted for by established individual-level risk factors.

## 2 Materials and methods

### 2.1 Study area

The study area is located in Hlabisa sub-district, one of the five sub-districts in rural Umkhanyakude in northern KwaZulu-Natal, South Africa. The area is 438 km^2^ in size and has a total population of approximately 90,000 people from approximately 11,000 households, of whom at any point of time about 65,000 are resident in the area. The area is predominantly rural but contains an urban township (where the socio-economic status is higher) and high-density settlements located along the national road running along its eastern boundary. The majority of its population lives in scattered homesteads not concentrated into any formal villages or compounds. The study area is typical of many rural South African settings (Tanser et al., 2008).

### 2.2 Study population

A large population-based survey measuring BMI and blood pressure was conducted in rural Umkhanyakude in KwaZulu-Natal, South Africa, a community with very high HIV prevalence, in 2003–2004 (Welz et al., 2007). Demographic and socioeconomic data were available from the Demographic Information System at the Africa Centre for Health and Population Studies, a Wellcome Trust-funded research centre at the University of KwaZulu-Natal. We got the information on HIV status from the annual HIV surveillance, which started in 2003. In the first round of the HIV surveillance, additional information was collected on weight, height, and blood pressure (Barnighausen et al., 2008).

The eligible population for this study consisted of all female residents aged 15–49 years and all male residents aged 15–54 years living in the surveillance area on June 18th, 2003. Residents are those reported living within the household. About 30% of all household members were not residents, i.e., they mainly lived elsewhere but still maintained connections with their households through periodic visits. As a pilot study to explore the effect of HIV infection on body mass and blood pressure, we were not able to measure body mass and blood pressure of the entire population because of limited resources at the time of data collection. As a result, one-third of sub-areas taken from all of the social, cultural and physical landscapes in the study area were reached for measurement of body mass and blood pressure.

All measurements followed the WHO STEPS protocol (WHO, 2003). BMI was calculated as an individual's body weight (in kilograms) divided by the square of his or her height (in meters). Those who have a BMI≥25 are categorized into the group of overweight. Three measurements of blood pressure were taken on the right arm at time intervals of at least 5 min. The average of the second and the third readings was used to estimate systolic and diastolic blood pressure (SBP, DBP). We used recent definitions of hypertension (stage-I, SBP≥140 mmHg or DBP≥90 mmHg). Seventy-three per cent of the 4896 eligible residents were successfully contacted. Valid measurements of height, weight, and SBP and DBP were obtained from 2311, 2350, 2307 and 2316 eligible residents, respectively.

This surveillance had ethical approval from the University of KwaZulu-Natal, Durban, South Africa. All participants gave their informed consent and no incentives were provided to participants.

### 2.3 Statistical analysis

To describe the distribution of basic demographic variables, *t*-tests were used for the continuous variables and *Χ*^2^ tests were used for categorical variables. Two groups of unconditional logistic regression models both for overweight and hypertension (SBP≥140 mmHg or DBP≥90 mmHg) were constructed adjusting for well-known determinants. These covariates included sex, 5-year age groups, educational attainment, household wealth quintile, marital status (currently married/coupled vs. single), place of residence (rural vs. urban/peri-urban residence), and HIV status. Previous research showed that age, sex, family wealth, marital status, place of residence, HIV status were significantly associated with BMI, and with either SBP or DBP in this study population (Barnighausen et al., 2008). Education attainment was also adjusted in the regression models as a covariate because it was known to be an important determinant of obesity, hypertension, and other cardiovascular diseases in the South African population (Kimani-Murage et al., 2011; Seedat et al., 1982). As a measure of household wealth, an assets index was developed using principal component factor analysis on living conditions (household ownership, access to piped water, access to electricity, main energy source for cooking, toilet type) and 27 household assets. Households were then categorized into quintiles on the assets index scale. Educational attainment was divided according to the main categories of the South African education system, which are no schooling, primary school (school years 1–7) and secondary school or higher (above school year 7). HIV status as assessed in HIV surveillance (positive, negative, not measured) was also added. To examine the effect of overweight on hypertension, an additional model was constructed for hypertension with overweight (BMI≥25 vs. BMI<25) included as an adjusted covariate.

### 2.4 Spatial analysis

**Fig. 1:**
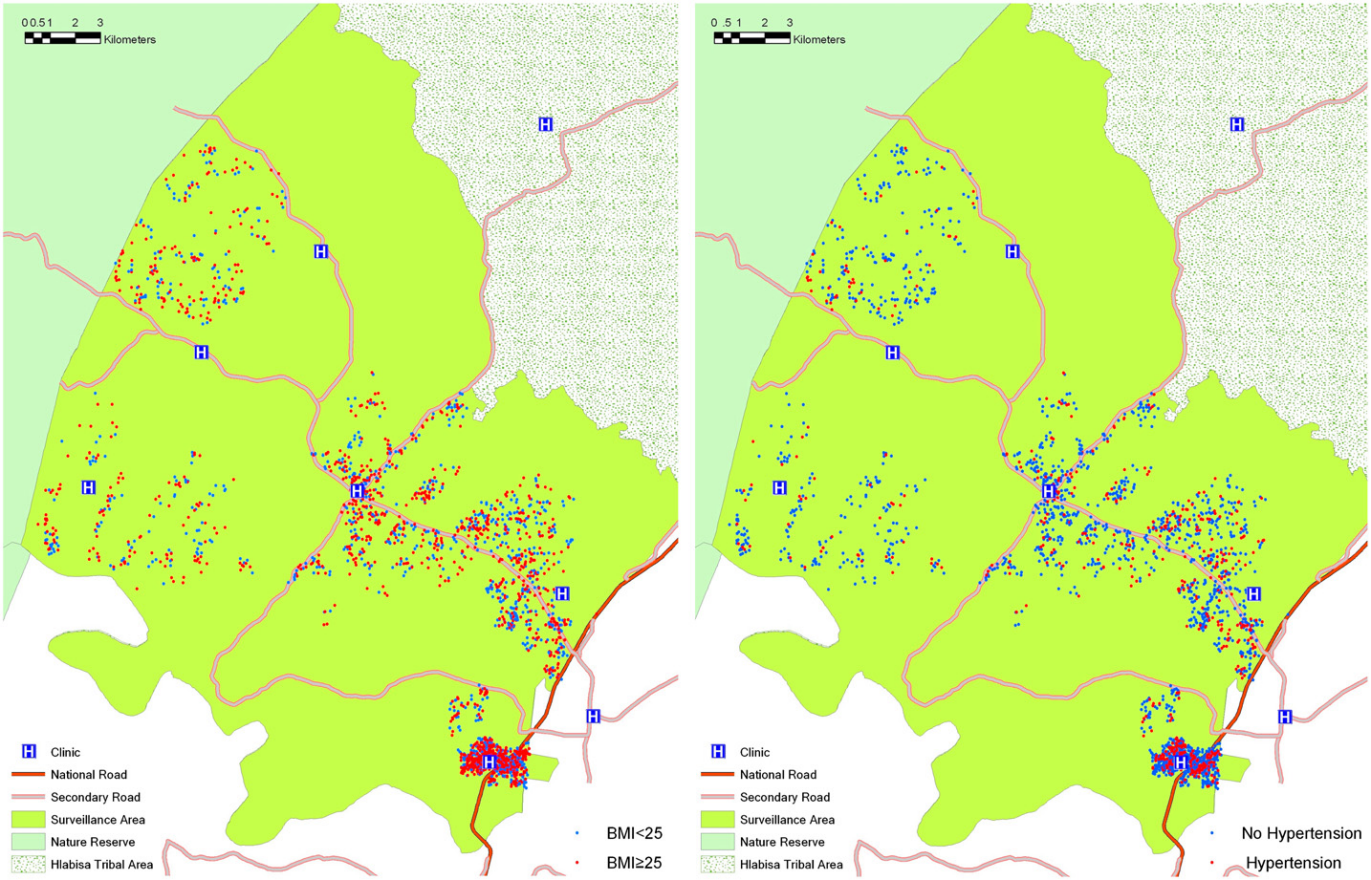
Maps of the study area showing the approximate location of participants. Participants were coded according to overweight status (left) and hypertension status (right).

All participants were geo-located to their homestead of residence by field workers using differential global positioning systems to an accuracy of <2 m (Tanser et al., 2001) (Fig. 1). A small random error was incorporated into the geographical position of each participant's home address before the spatial analysis. Because a single homestead with one geographical position often accommodated multiple adults, a small random error moved them slightly out of one household into different points per individual, which created a clear visual representation of the distribution of all participants. In addition, the small random error served as a method to protect confidentiality of the participants.

Spatial Scan Statistic (SaTScan) version 8.1.1 (National Cancer Insitute, Bethesda, MD, USA, www.satscan.org) was used in this study to detect cluster of high or low prevalence of overweight or hypertension. SaTScan uses the Kulldorff scan statistic to analyze spatial, temporal, and space-time point data (Kulldorff, 1997, 2006; Kulldorff et al., 2005). A spatial scan statistic is a cluster detection test able to detect the location of clusters, if any, and then evaluate their statistical significance, which is done by gradually scanning a window across space. The scan statistic can adjust for the uneven geographical density of a background population and the analysis is conditioned on the total number of cases observed. The spatial scan statistic imposes a circular window on the map and it allows the center of the circle to move across the entire study area gradually. For any given location of the center of the circle, the radius is changed continuously so that it can take any value from 0 up to a pre-specified maximum value (Kulldorff et al., 2005).

A Bernoulli probability model was used to identify spatial clusters of high and low prevalence in the distribution of overweight or hypertension. For each potential cluster, a likelihood ratio test was applied to test the null hypothesis of absolute spatial randomness against the alternative hypothesis that the probability of being a case in this circle was more or less than the probability of being a case outside. The *p*-value of the statistic was obtained through Monte Carlo hypothesis testing (9999 iterations), where the null hypothesis was rejected if the simulated *p*-value was less than 0.05. The maximum search radius of the circle was set to be 3 km, which was considered as the estimated size of a geographic community in our study area. The SaTScan output included the geographic location of the center, the radius, the observed and expected number of cases, the total population, the relative risk (RR), and the *p*-values of all of the identified clusters. In addition, we analyzed the standardized Pearson residuals from the statistical analysis outlined above in SaTScan using a normal model to identify the location of clusters after adjustment of potential confounders. For each potential cluster, a likelihood ratio test was applied to test the null hypothesis that all observations come from the same normal distribution against the alternative hypothesis that there was one cluster location where the observations had a larger mean than outside that cluster. This was accomplished by evaluating the statistical significance of clusters through a permutation based Monte Carlo hypothesis testing procedure (9999 iterations).

## 3 Results

Valid BMI and blood pressure measurements were obtained from 2298 and 2307 participants respectively. Fig. 1 shows the spatial distribution of all participants coded by overweight (left) and hypertension (right) status. A summary of subject characteristics by overweight and hypertension status is presented in Table 1. Among the 2298 participants with both valid height and weight measurement, 1341 (58.4%) were overweight, and among the 2307 participants with valid SBP and DBP, 522 (22.6%) were hypertensive. Overweight subjects were more likely to be female (83.67% vs. 45.98%, *p*<0.001) and live in urban or peri-urban areas (49.59% vs. 43.16%, *p*=0.002), compared to subjects who were not overweight. Relative to subjects who were not hypertensive, hypertensive subjects were older (42.2 vs. 38.2 years, *p*<0.001) and more likely to be male (35.63% vs. 30.48%, *p*=0.03), less likely to attain a secondary or higher education (44.55% vs. 55.17%, *p*=0.001), and belonged to the wealthiest household wealth quintile (20.51 vs. 26.64%, *p*=0.007). Overweight subjects had significantly lower prevalence of positive HIV (31.7% vs. 44.1%, *p*<0.001, among the subset of 1412 people with both BMI and HIV status available).

Two groups of logistic models for overweight and hypertension were constructed and the results are summarized in Table 2. Older age, female sex, secondary or higher educational attainment, and living in urban or peri-urban areas were all positively associated with being overweight (all *p*<0.05). Hypertension status was only found to be associated with older age. To investigate the effect of HIV status on overweight and hypertension, we further added HIV status to the regression models. The results are summarized in Table 2. Holding other factors constant, subjects with positive HIV status had significantly lower prevalence of overweight compared with those with negative HIV status (OR: 0.46, 95% CI: 0.35, 0.62). Subjects with positive HIV status also had lower prevalence of hypertension, but not statistically significant (OR: 0.84, 95% CI: 0.61, 1.17).

**Fig. 2:**
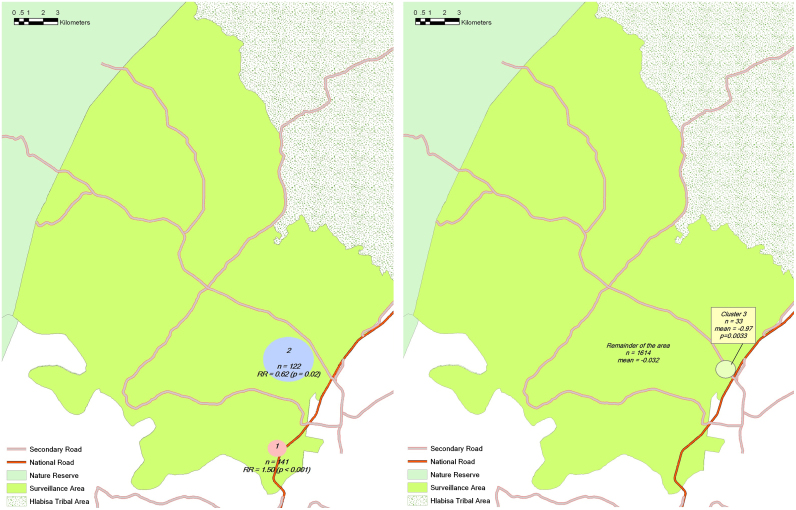
Geographic clusters of high prevalence and low prevalence of overweight indentified by SaTScan. Left: cluster 1 of high prevalence of overweight was identified using the Bernoulli model as the most likely cluster. This cluster was found to be in the only urban township in the surveillance area next to the national road. Additionally, cluster 2 was identified as low-risk area, in the nearby high-density settlement area. Both clusters were unlikely to have arisen by chance alone. Right: cluster of low mean value of standardized Pearson residuals of overweight model after adjusting for sex, 5-year age groups, educational attainment (no schooling, primary school and secondary school or higher), family wealth quintiles, marital status (married/coupled vs. single), and rural vs. urban residence.

One cluster of high prevalence of overweight (cluster 1: RR=1.50, *p*=0.0001) was identified by the SaTScan using the Bernoulli model as the most likely cluster (Fig. 2, left). This high-prevalence cluster was found to be in the only urban township in the surveillance area next to the national road. Additionally, one secondary low-prevalence cluster was identified, in the nearby high-density settlement area (cluster 2: RR=0.62, *p*=0.021) (Fig. 2, left). Both clusters were unlikely to have arisen by chance alone. Table 3 shows the basic characteristics of the two identified clusters. The prevalences of overweight in these two clusters were 85.1% and 36.9%. The high-prevalence area was characterized by higher aggregate level of education in comparison to the low-prevalence area. However, mean of age, household wealth, current marital status, and HIV prevalence were not significantly different.

After controlling for various risk factors including sex, 5-year age group, educational attainment, family wealth quintile, marital status, and place of residence, the spatial pattern of overweight clusters changed. Not only did the above two clusters disappear, but SaTScan identified another cluster with significantly lower mean value of standardized Pearson residuals (cluster 3, *p*<0.05). The center of the cluster was located right next to the national road (Fig. 2b). After further controlling HIV status in the model, the above cluster of lower mean value of residuals was no longer identified by SaTScan.

We used the same methods to search for spatial clusters of hypertension in the study area. There were no statistically significant clusters for hypertension before or after controlling for risk factors.

## 4 Discussion and conclusions

To our knowledge, our work is the first to investigate both determinants and spatial patterns of overweight and hypertension in a rural South African population with high HIV prevalence. Our findings show that the prevalence of overweight and hypertension was 58.4% and 22.6% in this population, respectively. We detected the existence of a strong spatial pattern in overweight prevalence in the study area. These findings take forward the results of a previous study published on the results of this large population-based survey measuring BMI and blood pressure which focused on the individual-level determinants of overweight and hypertension (Barnighausen et al., 2008).

The incidence of new HIV infection remains high in this rural South African community with high HIV prevalence (Bärnighausen et al., 2009). Previous work in this population found that people who tested HIV positive in the surveillance had significantly lower BMI than those who tested negative (Barnighausen et al., 2008). Our regression model confirmed that HIV positive subjects had significantly lower odds of being overweight compared to HIV negative subjects (OR: 0.46, 95% CI: 0.35, 0.62). We further found that the low-prevalence area of overweight had higher HIV prevalence, compared to the high-prevalence area of overweight (41.58% vs. 30.65%), although the difference was not statistically significant. The high-prevalence area of overweight, which was found to be in the only urban township in the surveillance area, was characterized by much higher level of education (80.20% with secondary school or higher education) compared to the low-prevalence area (47.57%) or the remainder of the study area (51.86%). After adjusting for important determinants, another small cluster of low overweight prevalence characterized by high HIV prevalence was identified, but the two clusters identified earlier disappeared. This suggested that the majority of the geographic variation of overweight prevalence may be accounted for by these known risk factors.

Previous studies suggested that common socioeconomic determinants, such as education, income, occupation, and urban or rural dwelling, are associated with hypertension (Grotto et al., 2008). It is believed that the awareness of hypertension prevention and the accessibility and adherence to medical control are higher among people with higher socioeconomic status. However, in our regression models, socioeconomic status was not found to be related to hypertension, which may explain the absence of clusters of hypertension in this area.

The application of spatial analysis to explore the geographic distribution of overweight and hypertension has mostly been limited to the developed world. The technology has been applied to detect differences in the prevalence of obesity and self-reported type 2 diabetes and in medical practice patterns among metropolitan statistical areas in the United States (Ford et al., 2005). In Canada, the technology has been used to reveal overweight and obesity clusters and underscore the importance of geographically focused prevention strategies informed by population-specific needs for the purpose of targeting scarce public health resources (Pouliou and Elliott, 2009). And in Leeds, UK, spatial analysis has been used to estimate determinants of childhood obesity (Edwards and Clarke, 2009). Striking geographic variations were found and public health intervention programs could be designed to specifically target populations living in high risk communities. However, this is the first time spatial analysis has been used to explore clustering of overweight and hypertension in a rural area of a region characterized by limited access to economic and health care resources and high levels of HIV prevalence.

An important strength of our approach is that we do not aggregate the data by arbitrary administrative units but instead use the precise location of each individual in the clustering analysis. However, the spatial methods used have several limitations. First of all, clusters are all arbitrarily defined as pure circles in the Kulldorff spatial scan statistic. Moreover, although the full range of human landscapes were covered in this area in the third of sub-areas sampled, the lack of complete coverage makes it likely that we failed to detect significant clustering in some communities of the study area which were not sampled.

In summary, our study provided clear evidence for the localized clustering of overweight, higher or lower than the expected proportion, but no spatial clustering of hypertension was identified. There are numerous physical, socioeconomic, nutritional, and cultural factors that correlate with each other to cause people with or without overweight to cluster in a neighborhood. In order to fully understand the reasons for the geographic variations in overweight prevalence in this population, the follow-up study needs to collect the information of some other important determinants, such as physical activity, access to transportation, and occupation. Additional specific factors that may contribute to the emergence of chronic diseases, including levels of dietary westernization, closeness to grocery stores, and health awareness, need to be considered in the future research.

## Figures and Tables

**Table 1:** Distribution of descriptive characteristics for the study population, according to overweight and hypertension conditions.

Characteristic	No. of overweight (*n*=957)^a^	Overweight (*n*=1341)^a^	*p*	No hypertension (*n*=1785)^b^	Hypertension (*n*=522)^b^	*p*
Age, mean±SD	38.83±7.0	39.20±6.5	0.20	38.2±6.6	42.2±6.2	<0.001

Sex, *n* (%)			<0.001			0.03
Male	517 (54.02)	219 (16.33)		544 (30.48)	186 (35.63)	
Female	440 (45.98)	1122 (83.67)		1241 (69.52)	336 (64.37)	

Educational attainment, *n* (%)			0.54			0.001
No schooling	116 (14.81)	147 (13.52)		192 (13.22)	73 (17.30)	
Primary school	262 (33.46)	351 (32.29)		459 (31.61)	161 (38.15)	
Secondary/higher	405 (51.72)	589 (54.19)		801 (55.17)	188 (44.55)	

Family wealth quintile, *n* (%)			0.86			0.007
Poorest quintile	115 (13.67)	176 (14.81)		222 (14.11)	67 (14.32)	
2nd Quintile	138 (16.41)	180 (15.15)		225 (14.30)	96 (20.51)	
3rd Quintile	192 (22.83)	260 (21.89)		350 (22.25)	104 (22.22)	
4th Quintile	189 (22.47)	269 (22.64)		357 (22.70)	105 (22.44)	
Wealthiest quintile	207 (24.61)	303 (25.51)		419 (26.64)	96 (20.51)	

Marital status, *n* (%)			0.78			0.38
Single/separated	652 (68.78)	906 (68.22)		1201 (67.85)	360 (69.90)	
Married/coupled	296 (31.22)	422 (31.78)		569 (32.15)	155 (30.10)	

Place of residence, *n* (%)			0.002			0.55
Rural	544 (56.84)	676 (50.41)		953 (53.39)	271 (51.92)	
Urban/peri-urban	413 (43.16)	665 (49.59)		832 (46.61)	251 (48.08)	

HIV status, *n* (%)			<0.001			0.115
Negative	348 (36.36)	539 (40.19)		671 (37.59)	221 (42.34)	
Positive	275 (28.74)	250 (18.64)		421 (23.59)	107 (20.50)	
Missing	334 (34.90)	552 (41.16)		693 (38.82)	194 (37.16)	

Abbreviation: SD, standard deviation.

a Overweight: body mass index≥25; no overweight: body mass index<25.

b Hypertension: SBP≥140 mmHg or DBP≥90 mmHg; no hypertension: SBP≤140 mmHg and DBP≤90 mmHg.

**Table 2:** Multiple logistic regression results: determinants of overweight and hypertension.

	Overweight^a^		Hypertension^b^	
	Model 1.1	Model 1.2	Model 2.1	Model 2.2
	OR (95% CI)	OR (95% CI)	OR (95% CI)	OR (95% CI)
Age (5-year groups)	1.28 (1.18, 1.40)	1.25 (1.14, 1.37)	1.60 (1.45, 1.77)	1.59 (1.44, 1.76)

Sex				
Male	1.00 (Referent)	1.00 (Referent)	1.00 (Referent)	1.00 (Referent)
Female	7.29 (5.68, 9.36)	7.64 (5.92, 9.85)	0.97 (0.74, 1.26)	0.97 (0.74, 1.26)

Education				
No schooling	1.00 (Referent)	1.00 (Referent)	1.00 (Referent)	1.00 (Referent)
Primary school	1.17 (0.83, 1.64)	1.21 (0.86, 1.71)	0.99 (0.70, 1.42)	1.00 (0.70, 1.42)
Secondary or higher	1.69 (1.21, 2.37)	1.66 (1.18, 2.35)	0.96 (0.67, 1.38)	0.96 (0.67, 1.38)

Household wealth				
Poorest quintile	1.00 (Referent)	1.00 (Referent)	1.00 (Referent)	1.00 (Referent)
2nd Wealth quintile	0.83 (0.56, 1.24)	0.85 (0.57, 1.27)	1.39 (0.91, 2.10)	1.40 (0.92, 2.12)
3rd Wealth quintile	0.88 (0.61, 1.27)	0.95 (0.65, 1.38)	0.83 (0.55, 1.24)	0.84 (0.56, 1.26)
4th Wealth quintile	0.94 (0.64, 1.36)	0.99 (0.68, 1.43)	0.90 (0.60, 1.34)	0.90 (0.60, 1.35)
Wealthiest quintile	0.96 (0.66, 1.38)	1.02 (0.70, 1.48)	0.71 (0.47, 1.07)	0.71 (0.48, 1.07)

Marital status				
Single	1.00 (Referent)	1.00 (Referent)	1.00 (Referent)	1.00 (Referent)
Married/coupled	0.98 (0.77, 1.24)	0.95 (0.75, 1.21)	0.90 (0.69, 1.17)	0.90 (0.69, 1.17)

Place of residence				
Rural	1.00 (Referent)	1.00 (Referent)	1.00 (Referent)	1.00 (Referent)
Urban/peri-urban	1.37 (1.10, 1.72)	1.42 (1.13, 1.78)	1.19 (0.92, 1.52)	1.19 (0.93, 1.53)

HIV status				
Negative		1.00 (Referent)		1.00 (Referent)
Positive		0.46 (0.35, 0.62)		0.84 (0.61, 1.17)
Missing		1.00 (0.78, 1.29)		0.97 (0.74, 1.27)

Overweight status				
Log likelihood	−974.2	−957.1	−830.0	−829.4
Pseudo *R*-squared	0.130	0.145	0.069	0.070
*n*	1647	1647	1655	1655

a Overweight: body mass index≥25.

b Hypertension: SBP≥140 mmHg or DBP≥90 mmHg.

**Table 3:** Clusters with high and low prevalences of overweight indentified by SaTScan.

Characteristic	Cluster 1 (*n*=141)	Cluster 2 (*n*=122)	*p*-Value of difference
Description	Urban/high-density settlement	High-density settlement	
RR^a^	1.50	0.62	
*p*-Value	<0.001	0.021	
Radius (km)	0.58	1.54	
Overweight (%)	85.11	36.89	<0.001
Age (mean)	38.79	39.16	0.67
Currently married/coupled (%)	31.91	27.05	0.39
Secondary school or higher (%)	80.20	47.57	<0.001
Highest quintile family wealth (%)	27.50	29.25	0.16
Living in urban/peri-urban area (%)	100.00	63.11	<0.001
HIV prevalence (%, among the tested)	30.65	41.58	0.16

a RR: relative risk of being overweight among subjects in the clusters compared to subjects outside of the clusters.

## References

[bib1] Bärnighausen T., Tanser F., Newell M.L. (2009). Lack of a decline in HIV incidence in a rural community with high HIV prevalence in South Africa, 2003–2007. AIDS Research and Human Retroviruses.

[bib2] Barnighausen T., Welz T., Hosegood V., Batzing-Feigenbaum J., Tanser F., Herbst K., Hill C., Newell M.L. (2008). Hiding in the shadows of the HIV epidemic: obesity and hypertension in a rural population with very high HIV prevalence in South Africa. Journal of Human Hypertension.

[bib3] Beyers N., Gie R.P., Zietsman H.L., Kunneke M., Hauman J., Tatley M., Donald P.R. (1996). The use of a geographical information system (GIS) to evaluate the distribution of tuberculosis in a high-incidence community. South African Medical Journal.

[bib4] Black J.L., Macinko J. (2008). Neighborhoods and obesity. Nutrition Reviews.

[bib5] Bourne L.T., Lambert E.V., Steyn K. (2002). Where does the black population of South Africa stand on the nutrition transition?. Public Health Nutrition.

[bib6] Bradshaw D., Groenewald P., Laubscher R., Nannan N., Nojilana B., Norman R., Pieterse D., Schneider M., Bourne D.E., Timaeus I.M., Dorrington R., Johnson L. (2003). Initial burden of disease estimates for South Africa, 2000. South African Medical Journal.

[bib7] Cook I., Alberts M., Lambert E.V. (2008). Relationship between adiposity and pedometer-assessed ambulatory activity in adult, rural African women. International Journal of Obesity.

[bib8] Department of Health, Medical Research Council, OrcMacro (2007). South Africa Demographic and Health Survey 2003.

[bib9] Edwards K.L., Clarke G.P. (2009). The design and validation of a spatial microsimulation model of obesogenic environments for children in Leeds, UK: SimObesity. Social Science and Medicine.

[bib10] Ford E.S., Mokdad A.H., Giles W.H., Galuska D.A., Serdula M.K. (2005). Geographic variation in the prevalence of obesity, diabetes, and obesity-related behaviors. Obesity Research.

[bib11] Grotto I., Huerta M., Sharabi Y. (2008). Hypertension and socioeconomic status. Current Opinion in Cardiology.

[bib12] Hessel P.A. (1985). Hypertension in white South-African miners. South African Medical Journal.

[bib13] Kimani-Murage E.W., Kahn K., Pettifor J.M., Tollman S.M., Klipstein-Grobusch K., Norris S.A. (2011). Predictors of adolescent weight status and central obesity in rural South Africa. Public Health Nutrition.

[bib14] Kulldorff, M., 1997. A spatial scan statistic. Communications in Statistics—Theory and Methods 26, pp. 1481–1496.

[bib15] Kulldorff M., Heffernan R., Hartman J., Assuncao R., Mostashari F. (2005). A space-time permutation scan statistic for disease outbreak detection. PLoS Medicine.

[bib16] Kulldorff, M., Information Management Services, Inc., 2006. SaTScan v7.0: Software for the Spatial and Space-time Scan Statistics.

[bib17] Maepe L.M., Outhoff K. (2012). Hypertension in goldminers. South African Medical Journal.

[bib18] Mayosi B.M., Flisher A.J., Lalloo U.G., Sitas F., Tollman S.M., Bradshaw D. (2009). The burden of non-communicable diseases in South Africa. Lancet.

[bib19] Mollentze W.F., Moore A.J., Steyn A.F., Joubert G., Steyn K., Oosthuizen G.M., Weich D.J. (1995). Coronary heart disease risk factors in a rural and urban Orange Free State black population. South African Medical Journal.

[bib20] Mowafi M., Khadr Z., Bennett G., Hill A., Kawachi I., Subramanian S.V. (2012). Is access to neighborhood green space associated with BMI among Egyptians? A multilevel study of Cairo neighborhoods. Health & Place.

[bib21] Pouliou T., Elliott S.J. (2009). An exploratory spatial analysis of overweight and obesity in Canada. Preventive Medicine.

[bib22] Seedat Y.K., Seedat M.A., Hackland D.B. (1982). Biosocial factors and hypertension in urban and rural Zulus. South African Medical Journal.

[bib23] Suastika K. (2006). Update in the management of obesity. Acta Medica Indonesiana.

[bib24] Tanser F., Barnighausen T., Cooke G.S., Newell M.L. (2009). Localized spatial clustering of HIV infections in a widely disseminated rural South African epidemic. International Journal of Epidemiology.

[bib25] Tanser F., Hosegood V., Bärnighausen T., Herbst K., Nyirenda M., Muhwava W., Newell C., Viljoen J., Mutevedzi T., Newell M.L. (2008). Cohort profile: Africa Center Demographic Information System (ACDIS) and population-based HIV survey. International Journal of Epidemiology.

[bib26] Tanser F., Hosegood V., Benzler J., Solarsh G. (2001). New approaches to spatially analyse primary health care usage patterns in rural South Africa. Tropical Medicine and International Health.

[bib27] Tanser F., Wilkinson D. (1999). Spatial implications of the tuberculosis DOTS strategy in rural South Africa: a novel application of geographical information system and global positioning system technologies. Tropical Medicine and International Health.

[bib28] van Rie A., Beyers N., Gie R.P., Kunneke M., Zietsman L., Donald P.R. (1999). Childhood tuberculosis in an urban population in South Africa: burden and risk factor. Archives of Disease in Childhood.

[bib29] Vorster H.H. (2002). The emergence of cardiovascular disease during urbanisation of Africans. Public Health Nutrition.

[bib30] Welz T., Hosegood V., Jaffar S., Batzing-Feigenbaum J., Herbst K., Newell M.L. (2007). Continued very high prevalence of HIV infection in rural KwaZulu-Natal, South Africa: a population-based longitudinal study. AIDS.

[bib31] WHO, 2000. Obesity: Preventing and Managing the Global Epidemic. Report of a WHO Consultation. World Health Organization Technical Report Series 894, i–xii, pp. 1–253. 11234459

[bib32] WHO (2003). STEPS Field Manual Guidelines for Field Staff.

[bib33] Wilkinson D., Tanser F. (1999). GIS/GPS to document increased access to community-based treatment for tuberculosis in Africa. Geographic information system/global positioning system. Lancet.

[bib34] Yancey A.K., Kumanyika S.K., Ponce N.A., McCarthy W.J., Fielding J.E., Leslie J.P., Akbar J. (2004). Population-based interventions engaging communities of color in healthy eating and active living: a review. Preventing Chronic Disease.

